# Serum cortisol and insulin-like growth factor 1 levels in major depressive disorder and schizophrenia

**DOI:** 10.1038/s41598-023-28449-8

**Published:** 2023-01-20

**Authors:** Hiroshi Arinami, Yuichiro Watanabe, Yutaro Suzuki, Misuzu Tajiri, Nobuto Tsuneyama, Toshiyuki Someya

**Affiliations:** Department of Psychiatry, Niigata University Graduate School of Medical and Dental Sciences, 757 Asahimachidori-Ichibancho, Chuo-ku, Niigata, 951-8510 Japan

**Keywords:** Endocrinology, Neurology

## Abstract

The pathophysiology underlying major depressive disorder (MDD) and schizophrenia is related to endocrine system functions and includes changes in the blood levels of cortisol and insulin-like growth factor 1 (IGF-1). However, these hormones have not been investigated simultaneously in patients with MDD and schizophrenia. We investigated the differences in serum cortisol and IGF-1 levels among patients with MDD and schizophrenia and controls. We included 129 patients with MDD, 71 patients with schizophrenia, and 71 healthy volunteers. Blood tests were performed between 6:00 am and 11:00 am after fasting. Serum cortisol levels were significantly higher in patients with schizophrenia than in patients with MDD and controls. Serum cortisol levels were significantly higher in patients with MDD than in controls. Serum IGF-1 levels were higher in both patient groups than in controls, whereas there was no significant difference between patients with MDD and schizophrenia. Both cortisol and IGF-1 levels were positively correlated with the Hamilton Rating Scale for Depression score in patients with MDD, whereas cortisol level was positively correlated and IGF-1 level was negatively correlated with the Brief Psychiatric Rating Scale score in patients with schizophrenia. The differences in the level of these hormones suggest pathophysiological differences between these disorders.

## Introduction

Major depressive disorder (MDD) and schizophrenia are major mental disorders with a relatively high prevalence^1,2^. Numerous studies have shown that hyperactivity of the hypothalamic–pituitary–adrenal (HPA) axis leads to reduced neurogenesis and synaptic plasticity in the hippocampus, which contributes to the pathophysiology of MDD and schizophrenia^3–11^. Moreover, several studies, including meta-analyses, have shown that blood cortisol levels are higher in patients with MDD and schizophrenia than in controls^3–6^. However, the differences in blood cortisol levels between MDD and schizophrenia have not been extensively investigated. Several studies have compared cortisol levels between patients with MDD and schizophrenia^12–16^, although results are inconsistent. The discrepancies in findings may be attributed to small sample sizes^12–16^ and the effects of confounders (e.g., age, sex, body mass index [BMI], diet, and comorbidity of other physical and psychiatric disorders)^8,17–19^.

Insulin-like growth factor-1 (IGF-1) has also been suggested to be involved in the pathophysiology of MDD and schizophrenia^20,21^. IGF-1 is mainly produced in the liver and crosses the blood–brain barrier to act on neurogenesis, synapse formation, myelination, and dendrite branching^20–24^. Preclinical studies have reported that IGF-1 induces antidepressant-like effects^25^ and that an increase in IGF-1 reduces cuprizone-induced demyelination and oligodendrocyte loss in a schizophrenia mouse model^26^. These findings suggest that IGF-1 is associated with the pathophysiology of MDD and schizophrenia. Several studies, including meta-analyses, have shown that patients with MDD and schizophrenia have higher blood IGF-1 levels than controls^21–24,27,28^. However, to date, no studies have verified the differences in blood IGF-1 levels between patients with MDD and schizophrenia. IGF-1 levels are affected by age, sex, BMI, other complications, duration of illness, and severity of symptoms^21–24,29–35^.

Several studies have reported an interaction between cortisol and IGF-1^36–38^. Cortisol inhibits IGF-1 synthesis in rat and sheep skeletal cells^37,38^ and modulates IGF-I release under conditions of fetal stress^36^. However, few studies have investigated the relationship between cortisol and IGF-1 in patients with MDD and schizophrenia, and the results have been inconsistent^24,35,39^. One study reported a nonsignificant positive trend in the correlation between the magnitude of decrease in IGF-I levels and the magnitude of decrease in cortisol levels in 78 patients with MDD during 6 weeks of antidepressant treatment^39^. Another study found a significant positive correlation between the magnitude of increase in IGF-1 levels and the magnitude of decrease in cortisol levels in 33 antipsychotic-naïve patients with schizophrenia during 3 months of antipsychotic treatment^35^. In contrast, cortisol levels were not significantly correlated with IGF-1 levels in 78 participants, including drug-naïve patients with schizophrenia and controls^24^. Therefore, further studies with simultaneous measurement of these hormones are necessary to investigate the pathogenesis of MDD and schizophrenia.

Our previous studied revealed that IGF-I and cortisol levels were associated with HAM-D scores in 91 patients with MDD^40^, and that serum IGF-1 levels were significantly higher in 54 male patients with MDD than in 37 male controls^41^. However, patients with schizophrenia were not included in our previous studies, and cortisol and IGF-I have not been investigated simultaneously in patients with MDD and schizophrenia. The present study extended the observation period and recruited additional participants aiming to determine the differences in serum cortisol and IGF-1 levels between patients with MDD and schizophrenia, while controlling for confounding factors, such as fasting, comorbidities, age, sex, and BMI. We also aimed to investigate whether the correlation between the levels of these hormones and symptom severity differs between the two disorders.

The impairment of negative feedback mechanisms in response to increased cortisol is common in patients with schizophrenia and MDD^42,43^. The levels of glucocorticoid receptors (GRs) in the hippocampus, which are involved in the negative feedback mechanisms that respond to increased cortisol, are lower in schizophrenia patients than in MDD patients^43,44^. Thus, cortisol levels are assumed to be highest in patients with schizophrenia, followed by patients with MDD and controls. In addition, IGF-1 crosses the blood–brain barrier and has neuroprotective functions^21,25,26,45^. Therefore, we hypothesized that IGF-1 would commonly be increased to compensate for the deficits in neuroplasticity and myelin formation that underlie schizophrenia and MDD^10,32,44,46–48^. Furthermore, because stress and cortisol secretion are positively correlated^49,50^, we hypothesized that cortisol levels would be correlated with the symptom severity of MDD and schizophrenia and reflect the impairment of negative feedback mechanisms in response to the increased cortisol in the two disorders^42,43^. Moreover, IGF-1 levels may be correlated with the symptom severity of the two disorders to compensate for the decrease in neuroplasticity and myelination as symptoms worsen^51,52^.

## Methods

### Participants

We included 129 patients with MDD, 71 patients with schizophrenia, and 71 healthy volunteers aged 18–64 years. Patients with MDD and schizophrenia were recruited from December 2015 to April 2021 from Niigata University Medical and Dental Hospital, and healthy control individuals matched for age, sex, and BMI were recruited from the community.

As of February 2017, we had recruited 91 patients with MDD for an initial study, which showed serum cortisol and IGF-1 levels were associated with symptom severity in patients with MDD^40^. Subsequently, as of March 2020, we had enrolled 54 male patients with MDD and 37 healthy male volunteers in a second study, which found serum IGF-1 levels were significantly higher in patients than in controls^41^. These studies included a total of 107 patients with MDD and 37 healthy male volunteers. The current study extended the observation period and recruited additional participants including 71 patients with schizophrenia who were not included in our previous studies^40,41^. Of the participants in our previous studies, 84 patients (61 males and 23 females) with MDD and 35 healthy male individuals were included in the current study. Seventy-one patients (48 males and 23 females) with MDD overlapped between the current and initial^40^ studies, and 44 male patients with MDD and 35 healthy male controls overlapped between our current study and second study^41^. Twenty-three patients were excluded for the following reasons: 14 met the diagnostic criteria for a psychiatric comorbidity other than MDD (bipolar II disorder, personality disorder, major neurocognitive disorder, adjustment disorder, unspecified depressive disorder, other specified depressive disorder, or persistent depressive disorder), four did not meet the age range inclusion criteria, four were diagnosed with a concurrent physical illness, and one had missing data. Two healthy volunteers were excluded because of declaration of protocol non-compliance (smoking or exercise).

Patients were diagnosed with MDD or schizophrenia according to the criteria in the Diagnostic and Statistical Manual of Mental Disorders, 5th Edition (DSM-5), and those with concurrent diagnoses of other mental illnesses were excluded because of the possible impact on cortisol and IGF-1^8,17,20,21,53–55^.

Comorbid diagnoses were determined by the active diagnoses at the time of enrollment (including diagnoses under treatment). The diagnostic requirements for the MDD group included current depressive episodes or remissions during treatment. Diagnoses were made by at least two psychiatrists. Patients were individually treated by clinicians. Eligibility criteria for the control group were no history of psychiatric consultation or medical history that met DSM-5 diagnostic criteria.

None of the participants had any physical illness (e.g., endocrine disorders; autoimmune disorders; malignant tumors; heart, lung, kidney, gastrointestinal, or nervous disorders; or infections in the past 2 weeks) that could affect their hormone levels, and none of the participants were pregnant, breastfeeding, or taking steroids or birth control pills.

This research was approved by the Ethics Committee on Genetics of Niigata University and conducted in compliance with the Declaration of Helsinki. Before participating in the study, all participants provided verbal and written informed consent, and if the ability to consent was questionable, informed consent was obtained from the participant’s parent and/or legal guardian.

### Blood tests and clinical assessments

All participants underwent venipuncture via the forearm cubital vein in the Niigata University Medical and Dental Hospital at rest between 6:00 am and 11:00 am following an overnight fast. Excessive exercise, smoking, and stressful activities (e.g., work or study) prior to blood collection were prohibited, and the evaluator verbally verified compliance with these instructions. The collected serum samples were centrifuged at 4 °C and stored at − 80 °C. The serum samples were analyzed using SRL Inc. (Tokyo, Japan). Serum cortisol levels were measured by electrochemiluminescence immunoassay using Elecsys Cortisol II kits (Roche Diagnostics K.K., Tokyo, Japan), and serum IGF-1 levels were measured by radioimmunoassay using IGF-1 (Somatomedin C) IRMA “Dai-ichi” kits (Fujirebio Inc., Tokyo, Japan). Serum cortisol and IGF-1 levels of 7.07–19.6 µg/dL and 64–574 ng/mL, respectively, were considered normal.

At the time of blood sample collection, the height and weight of all participants were measured, and patients underwent psychiatric symptom evaluation. The Global Assessment of Function (GAF) scale^56^ was used to evaluate global functional status (i.e., social, psychological, occupational functioning, and symptom statuses) of patients with MDD and schizophrenia. Symptom severity of patients with MDD and schizophrenia were evaluated using the Hamilton Depression Rating Scale (HAM-D)^57^ and the Brief Psychiatric Rating Scale (BPRS), respectively^58^. The HAM-D consists of 17 items, with higher scores indicating greater severity of depressive symptoms^57^. The BPRS consists of 16 items, each of which was rated on a scale of 0 (no symptoms) to 6 (most severe)^58^. Duration of illness was determined by self-reporting of when symptoms started.

### Statistical analysis

Data normality was assessed visually using Q–Q plots and confirmed using Shapiro–Wilk’s tests, where appropriate. The three groups were compared using a one-way analysis of variance with Welch’s correction and Games–Howell post hoc tests. Categorical data were analyzed using chi-square tests. Correlations between age and cortisol and IGF-1 for each group were evaluated using Pearson’s correlation coefficient, and differences in cortisol and IGF-1 between men and women for each group were evaluated using unpaired *t* tests. To more rigorously adjust for confounding factors indicated in previous studies, analysis of covariance was performed using GAF, duration of illness^21,24^, age^6,17,22,31^, sex^6,17,22^, and BMI^6,17,19,22,29,33^ as covariates for comparisons of cortisol and IGF-1 levels between patients with MDD and schizophrenia. The correlations between cortisol and IGF-1 levels and HAM-D and BPRS scores were evaluated using Pearson’s correlation coefficient. To investigate the factors affecting HAM-D and BPRS scores, we performed multiple regression analyses with the forward–backward stepwise selection method using age, sex, BMI, cortisol level, IGF-1 level, total imipramine equivalent dose (for MDD) or total chlorpromazine equivalent dose (for schizophrenia), and duration of illness as independent variables. Imipramine and chlorpromazine equivalent doses were calculated using established conversion formulas^59^. The level of statistical significance was set to *p* < 0.05. Data analyses were performed using the Statistical Package for the Social Sciences (SPSS) version 25 (IBM Japan, Tokyo, Japan).

## Results

### Serum cortisol and IGF-1 levels in patients with MDD and schizophrenia and controls

**Figure 1:**
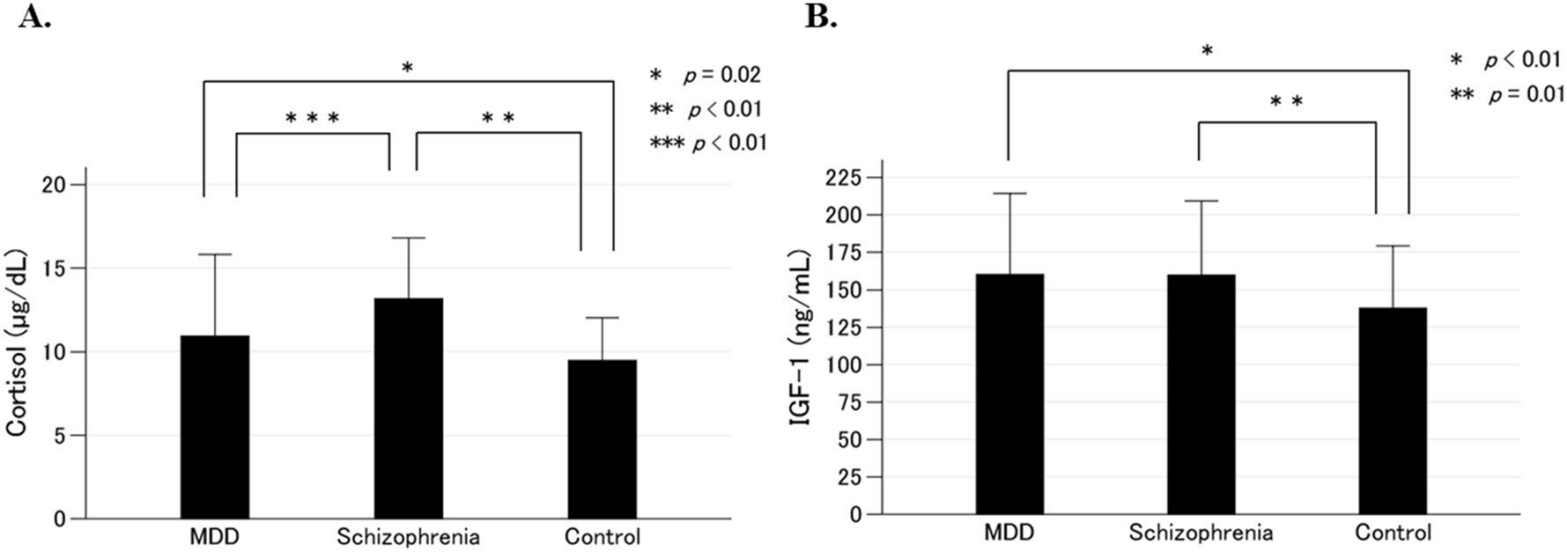
Serum levels of cortisol (**A**) and insulin-like growth factor 1 (IGF-1) (**B**) in patients with major depressive disorder (MDD), patients with schizophrenia, and controls. The mean and standard deviation for each group are shown as bar graphs and error bars, respectively. Serum cortisol levels in patients with MDD were higher than in controls (*p* = 0.02), and those in patients with schizophrenia were higher than in controls and patients with MDD (*p* < 0.01 and *p* < 0.01, respectively) (**A**). Serum IGF-1 levels were higher in patients with MDD and schizophrenia than in controls (*p* < 0.01 and *p* < 0.01, respectively). There were no significant differences in serum IGF-1 levels between patients with MDD and schizophrenia (*p* = 1.0) (**B**).

The clinical characteristics of the MDD, schizophrenia, and control groups are shown in Table 1. There were no significant differences in age, sex, or BMI among the three groups; however, there were significant differences in serum cortisol and IGF-1 levels among the three groups. Serum cortisol levels were significantly higher in patients with schizophrenia than in patients with MDD (*p* < 0.01) and controls (*p* < 0.01; Fig. 1A). Serum cortisol levels were significantly higher in patients with MDD than in controls (*p* = 0.02; Fig. 1A). Serum IGF-1 levels were significantly higher in patients with MDD (*p* < 0.01) and schizophrenia (*p* = 0.01) than in controls. There was no significant difference in serum IGF-1 levels between patients with MDD and schizophrenia (*p* = 1.0; Fig. 1B). The GAF score was significantly higher in patients with MDD than in those with schizophrenia (*p* < 0.01; Table 1). The duration of illness was significantly shorter in patients with MDD than in those with schizophrenia (*p* < 0.01; Table 1). When we excluded 44 male patients with MDD and 35 male controls in our previous study that found serum IGF-1 levels were significantly higher in patients than in controls^41^, the current results regarding cortisol and IGF-1 levels were not changed (Supplementary Table 1).

**Table 1:** Comparative profile of the major depressive disorder, schizophrenia, and control groups.

Variables	MDD	Schizophrenia	Control	*P-*value
Number	129	71	71	–
Age (years)^a^	40.5 ± 12.8	38.2 ± 9.9	41.4 ± 9.3	0.13^b^
Male/female	69/60	38/33	38/33	1.0^c^
Body mass index (kg/m^2^)^a^	22.6 ± 3.9	23.3 ± 3.9	23.2 ± 3.5	0.36^b^
Cortisol (µg/dL)^a^	11.0 ± 4.9	13.2 ± 3.6	9.5 ± 2.6	< 0.01^b^
IGF-1 (ng/mL)^a^	160.0 ± 54.3	159.7 ± 49.6	137.9 ± 41.3	< 0.01^b^
GAF^a^	39.3 ± 12.2	30.7 ± 9.1	–	< 0.01^d^
HAM-D^a^	13.9 ± 8.4	–	–	*–*
BPRS^a^	–	33.6 ± 9.5	–	–
Antidepressant drug use (%)	86.1	–	–	–
Imipramine equivalence (mg/day)^a^	125.0 ± 83.8	–	–	–
Antipsychotic drug use (%)	–	100	–	–
CP equivalence (mg/day)^a^	–	627.8 ± 353.6	–	–
Duration of illness (year)^a^	5.4 ± 6.2	14.8 ± 8.4	–	< 0.01^d^

^a^Data are expressed as means ± standard deviations.

^b^Analysis of variance with Welch’s correction.

^c^Chi-squared test.

^d^Unpaired *t* test.

*MDD* major depressive disorder, *IGF-1* insulin-like growth factor 1, *GAF* Global Assessment of Functioning, *HAM-D* Hamilton Rating Scale for Depression, *BPRS* Brief Psychiatric Rating Scale, *CP* Chlorpromazine.

Age was significantly positively correlated with serum cortisol levels in patients with MDD (R = 0.21, *p* = 0.02), but age and serum cortisol levels were not significantly correlated in patients with schizophrenia (*p* = 0.08) or controls (*p* = 0.05). Men had higher serum cortisol levels than women in patients with schizophrenia (*p* < 0.01) and controls (*p* < 0.01), whereas there were no significant differences between men and women in patients with MDD (*p* = 0.11; Supplementary Table 2). Age was significantly negatively correlated with serum IGF-1 levels in patients with MDD (R = − 0.54, *p* < 0.01) and schizophrenia (R = − 0.47, *p* < 0.01), and controls (R = − 0.55, *p* < 0.01). There were no significant differences in serum IGF-1 levels between men and women in patients with MDD (*p* = 0.47) or schizophrenia (*p* = 0.13), or controls (*p* = 0.23; Supplementary Table 2). Serum cortisol and IGF-1 levels were not significantly correlated in patients with MDD (R = − 0.80, *p* = 0.37) or schizophrenia (R = − 0.17, *p* = 0.16), or controls (R = 0.16, *p* = 0.18).

As mentioned earlier, a one-way analysis of variance with Welch’s correction and Games–Howell post hoc tests showed that serum cortisol levels were significantly higher in patients with schizophrenia than in patients with MDD (*p* < 0.01; Fig. 1A) and that there was no significant difference in serum IGF-1 levels between patients with MDD and schizophrenia (*p* = 1.0; Fig. 1B). Subsequently, we performed the analysis of covariance comparing serum cortisol and IGF-1 levels between patients with MDD and schizophrenia, using GAF, duration of illness, age, sex, and BMI as covariates. We confirmed that serum cortisol levels were significantly higher in patients with schizophrenia than in patients with MDD (*p* < 0.01). There was no significant difference in serum IGF-1 levels between patients with MDD and schizophrenia (*p* = 0.44).

### Serum cortisol and IGF-1 levels and symptom severity in patients with MDD and schizophrenia

**Figure 2:**
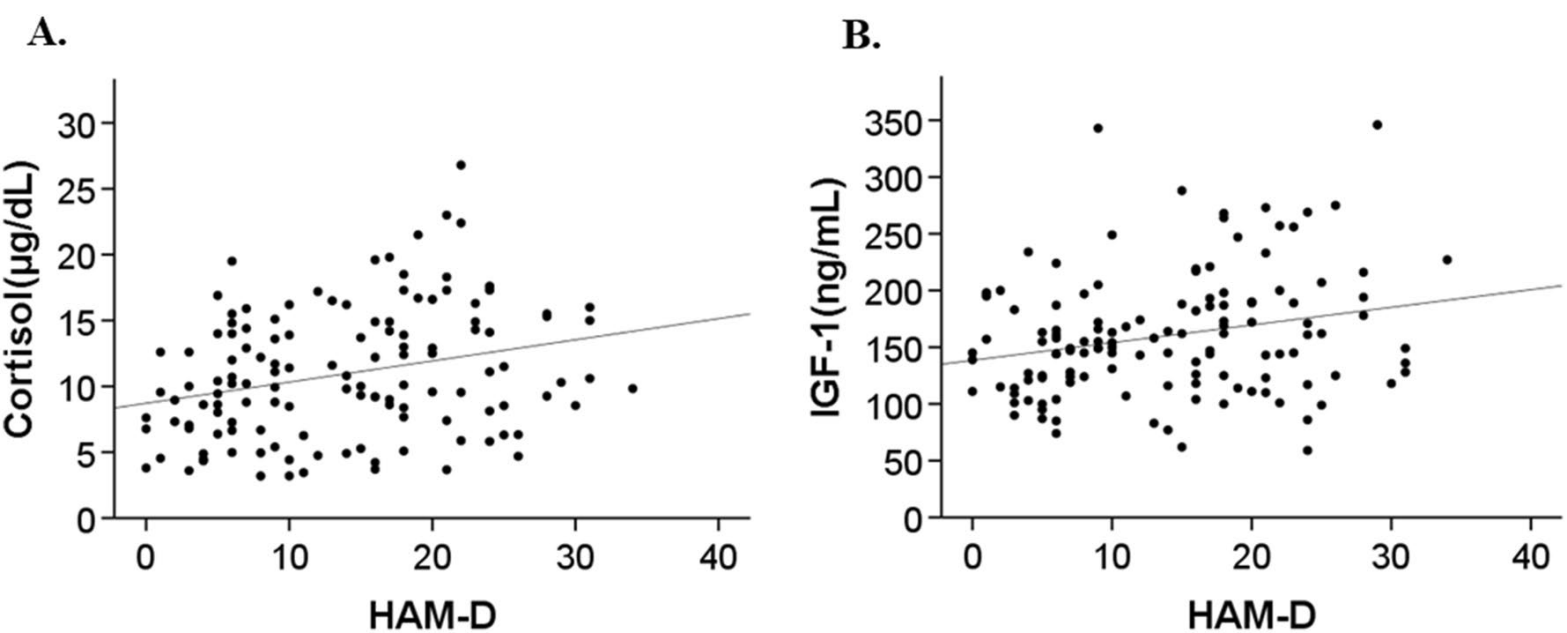
Scatterplots of serum cortisol level versus Hamilton Depression Rating Scale (HAM-D) score (**A**) and serum insulin-like growth factor (IGF)-1 level versus HAM-D score (**B**) in patients with major depressive disorder. Serum cortisol level was significantly positively correlated with HAM-D score (R = 0.28, *p* < 0.01) (**A**), and serum IGF-1 level was significantly positively correlated with HAM-D score (R = 0.24, *p* < 0.01) (**B**).

**Figure 3:**
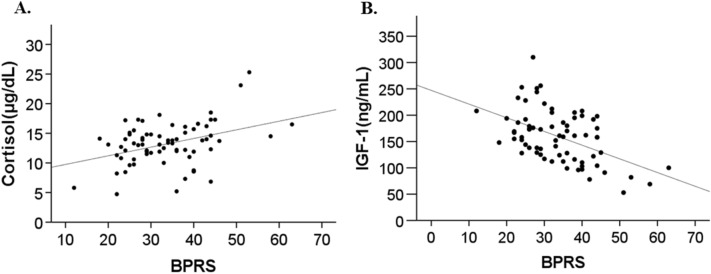
Scatterplots of serum cortisol level versus Brief Psychiatric Rating Scale (BPRS) score (**A**) and serum insulin-like growth factor (IGF)-1 level versus BPRS score (**B**) in patients with schizophrenia. Serum cortisol level was significantly positively correlated with BPRS score (R = 0.38, *p* < 0.01) (**A**), and serum IGF-1 level was significantly negatively correlated with BPRS score (R = − 0.50, *p* < 0.01) (**B**).

In patients with MDD, there were significant positive correlations between serum cortisol and IGF-1 levels and HAM-D scores (R = 0.28, *p* < 0.01 and R = 0.24, *p* = 0.01, respectively; Fig. 2). The total imipramine equivalent dose was not significantly correlated with serum cortisol (*p* = 0.64) or IGF-1 levels (*p* = 0.29). In patients with schizophrenia, we found a significant positive correlation between serum cortisol levels and BPRS scores (R = 0.38, *p* < 0.01; Fig. 3A) and a significant negative correlation between serum IGF-1 levels and BPRS scores (R = − 0.50, *P* < 0.01; Fig. 3B). The CP equivalent dose was not significantly correlated with serum cortisol (*p* = 0.15) or IGF-1 levels (*p* = 0.33).

Stepwise multiple regression analyses showed that higher serum cortisol and IGF-1 levels contributed to higher HAM-D scores (Table 2), whereas higher serum cortisol levels and lower serum IGF-1 levels contributed to higher BPRS scores (Table 3).

**Table 2:** Stepwise multiple regression analysis of the relationship between the Hamilton Rating Scale for Depression scores and independent variables.

Independent variables	Adjusted R^2^	B	SE	β	*P*
Cortisol	0.07	0.51	0.14	0.30	< 0.01
IGF-1	0.13	0.04	0.01	0.26	< 0.01

*IGF-1* insulin-like growth factor 1, *B* non-standardized regression coefficient B, *SE* standard error, *β* standardized regression coefficient beta.

**Table 3:** Stepwise multiple regression analysis of the relationship between the Brief Psychiatric Rating Scale scores and independent variables.

Independent variables	Adjusted R^2^	B	SE	β	*P*
Cortisol	0.32	0.80	0.26	0.31	< 0.01
IGF-1	0.24	− 0.09	0.02	− 0.44	< 0.01

*IGF-1* insulin-like growth factor 1, *B* non-standardized regression coefficient B, *SE* standard error, *β* standardized regression coefficient beta.

## Discussion

We found that serum cortisol levels were highest in patients with schizophrenia, followed by patients with MDD and controls. We also found that serum cortisol levels were positively correlated with symptom severity in patients with MDD and schizophrenia. In addition, the serum IGF-1 level of patients with MDD and schizophrenia was higher than that of controls. However, there was no significant difference in IGF-1 levels between the two patient groups. Furthermore, serum IGF-1 levels correlated positively with MDD symptom severity but negatively with schizophrenia symptom severity.

### Increased serum cortisol levels in patients with schizophrenia and MDD

Our study replicated previous findings that cortisol levels are significantly higher in patients with MDD and schizophrenia than in controls^3,4,6,7^. However, the results of previous studies comparing MDD and schizophrenia patients are inconsistent^12–16^, which may be because of the relatively small sample sizes of these studies (*n* = 44–91)^12–16^. In addition, the divergent results may be attributed to the confounding factors that are known to affect human cortisol levels^5,7,8,17–19,40,60^, which include age, sex, BMI, fasting status, endocrine and autoimmune physical comorbidities, psychiatric comorbidities, and the severity of psychiatric pathology^5,7,8,17–19,40,60^. Although cortisol levels in previous studies varied mainly by units of measurement and time of blood collection^12–16^, cortisol levels in our control group were generally consistent with those of previous studies that had the same units of measurement included in the meta-analysis^3^. Our sample size (*n* = 271) was larger than that of previous studies. Moreover, we adjusted for confounding factors that had not been considered in previous studies. As a result, we revealed for the first time that patients with schizophrenia have the highest levels of serum cortisol, followed by MDD patients and healthy controls.

Cortisol levels have been shown to correlate positively with the state of stress^49,50^. Thus, the higher cortisol levels in patients with schizophrenia may reflect a higher stress state in patients with schizophrenia, followed by MDD and controls. Postmortem brain studies have shown that GR expression in the hippocampus is lower in patients with schizophrenia than in patients with MDD^43^. Moreover, magnetic resonance imaging studies have shown that hippocampal volume is lower in patients with schizophrenia than in patients with MDD^44^. GRs suppress cortisol secretion via negative feedback on the HPA system^42^; thus, cortisol may be higher in patients with schizophrenia. Furthermore, given that peripheral cortisol secretion and brain dopamine release are positively correlated^61,62^, cortisol may be higher in schizophrenia patients with excessive dopamine secretion in the brain.

### Relationship between serum cortisol levels and symptom severity

In this study, we revealed a positive association between cortisol levels and symptom severity in both MDD and schizophrenia patients. This is consistent with the results of our previous study in MDD patients^40^ as well as the findings of other studies in MDD and schizophrenia patients^5,7,60^. This finding may be explained by several hypotheses. In both disorders, greater symptom severity may lead to a higher stress load, resulting in increased cortisol production. Cortisol can exacerbate impaired neuroplasticity, which is one of the prevailing hypotheses underlying the pathology of MDD^9,10^. Thus, elevated cortisol levels may increase the symptom severity of MDD patients via impaired neuroplasticity. In addition^11,46^, in patients with schizophrenia, increased dopamine may lead to higher cortisol levels, either through the exacerbation of symptoms or via direct interaction between the dopamine neurotransmitter system and the HPA axis^61,62^.

### Increased serum IGF-1 levels in patients with MDD and schizophrenia

Although serum IGF-1 levels were higher in patients with MDD and schizophrenia than in the control group, the difference between MDD and schizophrenia patients was not significant. To the best of our knowledge, this is the first study to compare serum IGF-1 levels among patients with MDD and schizophrenia and controls. We confirmed the findings of two meta-analyses that showed that IGF-1 levels are increased in patients with MDD^21,27^. IGF-1 levels were elevated in our Japanese patients with schizophrenia who had a mean illness duration of 14.8 years, whereas IGF-1 levels were not altered in Turkish patients with schizophrenia who had a mean illness duration of 11.8 years^31^ or Japanese patients with schizophrenia who had a mean illness duration of more than 10 years^33^. However, the patients in these previous studies had a higher BMI than the controls^31^ and comorbid diabetes mellitus (DM)^31,33^, which may decrease IGF-1 levels^29^. We matched groups for BMI and excluded patients with DM and showed that serum IGF-1 levels were higher in patients with schizophrenia than in controls. IGF-1 levels in the control group of the current study were consistent with those in another Japanese study^33^, but lower than those in a Turkish study^31^. In all studies, IGF-1 levels were measured by radioimmunoassay. Although previous studies did not provide information regarding the kit used, IGF-1 levels were measured at same company in the current and another Japanese studies.

MDD and schizophrenia are associated with impaired neuroplasticity and myelin dysplasia^10,46–48^. Because IGF-1 has neuroprotective effects, such as neurogenesis and myelination, an increase in IGF-1 may indicate a compensatory function for these disorders^25,26,32,45^. However, IGF-1 levels did not differ between patients with MDD and schizophrenia, which suggests that IGF-1 level is not a specific marker of MDD or schizophrenia.

### Relationship between serum IGF-1 level and symptom severity

We observed a positive correlation between IGF-1 levels and MDD symptom severity, which is consistent with the results of our and other previous studies^22,40^. The higher IGF-I levels in patients with MDD may reflect a compensatory mechanism that offsets impaired neurogenesis and results in an exacerbation of symptoms^22,25,28,45^. This hypothesis is supported by the results of several preclinical and clinical studies^22,25,28,45^.

We found a negative correlation between IGF-1 level and schizophrenia symptom severity, which is consistent with the results of several previous studies^23,33,35^. In patients with schizophrenia, those with greater symptom severity had lower IGF-I levels, which was in contrast to those with MDD. IGF-1 resistance is one possible explanation for this finding. Similar to the high insulin concentration that results from insulin resistance, IGF-1 levels may initially increase to compensate for IGF-1 resistance before gradually becoming depleted. In other words, we speculate that the compensation mechanism of IGF-1 is reflected in the presentation of mild symptoms; however, the level of serum IGF-1 decreases because this compensation diminishes as symptoms worsen.

### Interaction between cortisol and IGF-1

In the present study, there was no correlation between cortisol and IGF-1 levels in any of the groups. Consistent with the present results, a cross-sectional study found no significant correlation between IGF-I and cortisol in patients with MDD or schizophrenia or control subjects^35,39,63^. However, a longitudinal study examining antipsychotic treatment-induced changes in antipsychotic naïve patients reported a significant positive correlation between changes in cortisol and IGF-I levels in patients with schizophrenia^35^. These results suggest that IGF-1 and cortisol may not correlate cross-sectionally, but may correlate in response to drug-induced changes in patients with MDD and schizophrenia. However, further studies, including longitudinal studies, are needed to confirm this hypothesis.

### Limitations

First, because we conducted this study in patients who were receiving treatment, we could not rule out the effect of psychotropic drugs. In the current study, imipramine or CP equivalent dose was not significantly correlated with serum cortisol or IGF-1 levels in patients with MDD or schizophrenia. Earlier studies have reported inconsistent results regarding the effects of antidepressants and antipsychotics on serum cortisol and IGF-1 levels^21–23,35,60^. Nevertheless, the generalizability of our findings is limited to patients undergoing treatment. To directly examine the correlation between symptom severity and cortisol and IGF-1, future studies should analyze only drug-naïve/-free cases. Second, our interpretation of blood hormone levels was limited. A preclinical study demonstrated that peripherally administered cortisol crosses the blood–brain barrier and binds to both glucocorticoid and mineralocorticoid receptors in the brain^64^. Similarly, a study in rats with ischemic stroke showed that intravenously administered IGF-1 crosses the blood–brain barrier and exerts its neuroprotective effects through IGF-1 receptors in the brain^65^. These reports suggest that there is a parallel relationship between peripheral and central hormone levels. However, no studies in humans, including patients with MDD and schizophrenia, have simultaneously examined peripheral and central cortisol and IGF-1 levels. Therefore, the extent to which peripheral cortisol and IGF-1 levels reflect those of the central nervous system remains unclear, which is a significant limitation of the current study. Third, cortisol levels follow circadian rhythms. In the current study, blood was collected only once between 6:00 am and 11:00 am, which could be a confounding factor. Fourth, although patients with endocrine disease were not included in this study on the basis of an interview and hormone levels that were within the normal range in almost all patients, the possibility of endocrine disease could not be completely ruled out because urinary free cortisol and dexamethasone suppression tests and oral glucose tolerance tests were not performed. Fifth, cortisol and IGF-1 levels were within normal ranges in all three groups with the exception of cortisol levels in five patients with MDD and two patients with schizophrenia. Therefore, the clinical significance should be interpreted with caution. Sixth, we could not exclude the possibility that differences in cortisol levels may be due to interacting effects between stress reactivity to venipuncture and patient status^8^. Measurement of hair cortisol concentrations may overcome this methodological limitation^55^. Finally, although we excluded patients with comorbidities, patients with schizophrenia may also have depressive symptoms, and those with MDD may also have psychotic symptoms. In practice, many patients have comorbidities; therefore, studying patients with comorbidities will be important in future studies.

## Conclusion

Serum cortisol levels were higher in patients with schizophrenia than in those with MDD, and the direction of correlation between serum IGF-1 level and symptom severity was contrasting between patients with MDD and those with schizophrenia, although there was no correlation between cortisol and IGF-1 levels in any of groups. These results suggest that the differences in cortisol and IGF-1 levels represent pathophysiological differences between the two disorders. Further investigations in larger sample sizes, including longitudinal studies, are needed to test this hypothesis.

## Supplementary Information

Supplementary Table 1.

Supplementary Table 2.

## Data Availability

The data are restricted by the Ethics Committee on Genetics of Niigata University. The data administrator can be contacted via email: arinami7676@gmail.com.
